# Epigenetics and Early Development

**DOI:** 10.3390/jdb10020026

**Published:** 2022-06-16

**Authors:** Gokul Gopinathan, Thomas G. H. Diekwisch

**Affiliations:** TAMU Center for Craniofacial Research and Diagnosis, Texas A&M College of Dentistry, Dallas, TX 75246, USA; gopinath@tamu.edu

**Keywords:** epigenetics, DNA methylation, histone acetylation, histone methylation, chromatin remodeling enzymes

## Abstract

The epigenome controls all aspect of eukaryotic development as the packaging of DNA greatly affects gene expression. Epigenetic changes are reversible and do not affect the DNA sequence itself but rather control levels of gene expression. As a result, the science of epigenetics focuses on the physical configuration of chromatin in the proximity of gene promoters rather than on the mechanistic effects of gene sequences on transcription and translation. In the present review we discuss three prominent epigenetic modifications, DNA methylation, histone methylation/acetylation, and the effects of chromatin remodeling complexes. Specifically, we introduce changes to the methylated state of DNA through DNA methyltransferases and DNA demethylases, discuss the effects of histone tail modifications such as histone acetylation and methylation on gene expression and present the functions of major ATPase subunit containing chromatin remodeling complexes. We also introduce examples of how changes in these epigenetic factors affect early development in humans and mice. In summary, this review provides an overview over the most important epigenetic mechanisms and provides examples of the dramatic effects of epigenetic changes in early mammalian development.

## 1. Introduction

**Figure 1:**
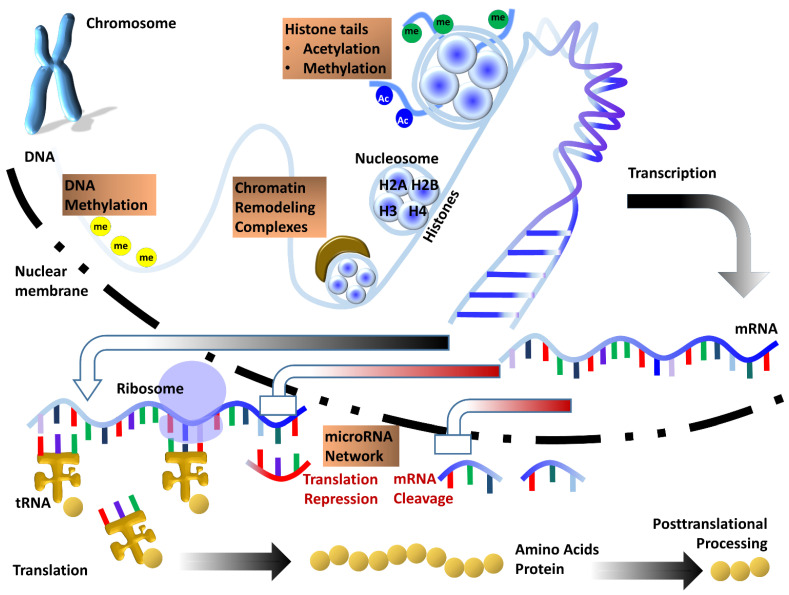
Sketch illustrating the multi-layered control of gene expression by various epigenetic processes. DNA is subject to direct chemical modification by addition or removal of methyl groups at cytosine bases. Epigenetic processes regulating gene expression at a higher level of DNA compaction include histone tail modifications and control of chromatin accessibility by ATP dependent chromatin remodeling complexes. Once transcribed, mRNA can be further regulated by the microRNA network which provides an additional layer of epigenetic control of gene expression. Together, these epigenetic mechanisms function in concert to help fine-tune gene expression within the cell.

Once dubbed a series of chemical tags that modify DNA and its associated structures, today’s view of the epigenome during development is that of an omnipotent control entity that shapes all stages of the development of an organism through mechanisms as diverse as DNA methylation, histone modifications, remodeling via ATP-dependent chromatin complexes, histone variant exchange, Polycomb complex mediated gene silencing, chromatin dynamics, heterochromatin effects on mitotic spindle anchorage, and interactions with environmental factors from nutrition to drugs and stress. While it is well accepted that epigenetics plays major roles in development, our understanding of the mechanisms involved and their effects on cellular differentiation and organ development is still in its infancy. How do individual epigenetic modifications control lineage specification and tissue specificity? How do various epigenetic mechanisms collaborate to exert control over cell fate commitment? What is the nature of the link between condensed heterochromatin and mitotic spindle formation at the onset of mitosis? What mechanisms govern chromatin dynamics to control cell fate? What happens to the organism if epigenetic control goes awry? Recent studies have been able to whet our appetite to understand the role of the epigenome in development, but there is much to learn, and this Special Issue is destined to explore and bridge some of our knowledge gaps (Figure 1).

The development of eukaryotic organisms not only depends on the sequence of individual DNA strands but also on the physical-chemical wrapping of DNA. Epigenetic modifications to the DNA are heritable and do not affect DNA itself. One prominent example of epigenetic modifications during development is the removal of DNA methyl marks through DNA demethylases in the hypomethylated epigenome of primordial germ cells and the preimplantation embryo. Following implantation, reestablishment of methylation results in a pattern of irreversible transcriptional silencing marks at select gene promoters that are stably propagated throughout mitosis [1,2]. A second mechanism of epigenetic regulation during development is provided by several histone tail modifications including acetylation, methylation, phosphorylation and ubiquitylation which are catalyzed by enzymes, including histone acetyltransferases, methyltransferases, deacetylases and demethylases. Common acetyl modifications occur at histone H4 (K5, K8, K12 and K16) and histone H3 (K9, K14, K18, K23 and K27) and affect transcriptional activation, while the most prominent histone lysine trimethyl marks (K4me3 versus K9me3 and K27me3) affect both gene activation and repression [3]. Another group of epigenetic modifiers that affect DNA accessibility during development and differentiation are the ATPase domain containing chromatin remodeling complexes, such as the SWI/SNF (switching defective/sucrose nonfermenting), CHD (chromodomain helicase DNA-binding), ISWI (imitation switch), and INO80 (inositol requiring 80) complexes that rely on ATP catalytic energy to exchange histones and to slide or evict nucleosomes [4]. The result of chromatin complex-mediated remodeling affects pluripotency, differentiation, and embryonic development.

## 2. DNA Methylation

A prominent and well understood example of epigenetic modification during development is the addition and removal of a methyl group at the C-5 position of the DNA cytosine ring by DNA methyltransferases and DNA demethylases dubbed writers and erasers, respectively. The writers of DNA methylation comprise the de novo DNA methyltransferases DNMT3A and DNMT3B and the maintenance methylase DNMT1, while active DNA demethylation is carried out by the ten-eleven translocation (TET) enzymes (TET1-3 in mammals) through oxidation of methylcytosine to hydroxymethylcytosine followed by a base excision repair pathway [5]. Eukaryotic DNA methylation is predominantly observed in the context of CpG dinucleotides in the genome and is observed at the promoters of virtually all housekeeping genes, developmentally regulated genes and genomic regulatory elements [6,7].

DNA methylation is associated with several key epigenetic processes such as genomic imprinting and X-chromosome inactivation (XCI), transposon silencing and aging [8]. The correlation between DNA methylation and transcription inhibition has been well established although exact mechanism of gene silencing has not entirely been resolved. Certain transcription factors exhibit decreased binding to their corresponding motifs on methylated DNA, impacting gene activation and transcription [9,10]. DNMTs also recruit chromatin remodelers and modifiers to methylated DNA, contributing to heterochromatin formation. In addition, methyl-CpG-binding domain (MBD) proteins (MBD1-MBD4), the methyl-CpG-binding protein 2 (MeCP2) and several zinc finger proteins recognize and directly bind methylated DNA, resulting in gene silencing [11,12]. As a result, DNA methylation directly controls the transcription and silencing of genes.

## 3. DNA Methylation in Development

During mammalian development, the epigenome is reprogrammed in two waves of global demethylation and remethylation events, one occurring during germ cell development and the other during early embryogenesis [13]. DNA methylation reprogramming during primordial germ cell specification is crucial for the erasure of somatic lineage epigenetic marks and for the establishment of new epigenetic marks, ensuring transmission of appropriate marks while avoiding inheritance of any potential epimutations in the embryo [13]. This process of DNA demethylation is believed to occur through an active, replication-independent mechanism involving components of the base excision repair pathway and TET enzymes [14,15,16,17]. New DNA methylation marks are then deposited in male and female primordial germ cells (PGC) by the de novo methyltransferases, DNMT3A together with the catalytically inactive DNMT3L, resulting in sex-specific germ cell methylation patterns [18,19,20]. Another phase of epigenomic reprogramming occurs in the preimplantation embryo shortly after fertilization, during which parental methylation marks inherited from both gametes are erased once more, with the exception of those at imprinted loci and some retrotransposons [1,21]. During this phase the paternal genome undergoes rapid replication-independent demethylation (active demethylation) while the maternal genome is passively demethylated [1,22]. Subsequently, upon implantation at the early blastocyst stage, the embryo is subject to another wave of *de novo* methylation events, restoring the initial embryonic methylation pattern [2]. This biphasic epigenetic reprogramming process during gametogenesis and embryogenesis is regulated by the DNA methylation writers and erasers mentioned above, the DNA methyltransferases DNMT1, DNMT3A, and DNMT3B and the demethylase TET3. Illustrating the essential role of DNA methyltransferases and demethylases for embryonic development in mammals, *Dnmt1^−/−^* and *Dnmt3b^−/−^* mice are embryonically lethal and exhibit extensive loss of DNA methylation, whereas *Dnmt3a^−/−^* mice are born severely runted and do not survive [23,24]. These findings are evidence of the essential role played by the DNA methylation and demethylation machinery during mammalian development. 

## 4. Histone Tail Modifications

The compact packaging of nuclear genomic material is facilitated by small basic histone proteins consisting of a central histone tetramer made of two H3:H4 dimers flanked on either side by H2A:H2B dimers that form a nucleosome core wrapped within 147 bp of DNA [25]. The histone proteins of the nucleosome core contain disordered N-terminal extensions, referred to as histone tails, which are subject to a plethora of posttranslational modifications, including methylation, acetylation, phosphorylation, ubiquitylation, sumoylation [3]. Although histone modifications were identified in the 1960s, their molecular function in the regulation of epigenetic processes has only been recently characterized and is a topic of several ongoing investigations [3,26].

Posttranslational histone modifications act as binding sites for other non-histone proteins and primarily influence gene expression by directly affecting chromatin compaction and accessibility of transcription factors to gene promoters [25]. Histone modification exert powerful control over gene expression through their association with either transcriptionally active or silent chromatin. For example, chromatin from promoter region of active genes exhibit high levels of trimethylation of histone H3 at lysine 4 (H3K4me3), lysine 36 (H3K36me3) and lysine 79 (H3K79me3), acetylation of lysine 9 in H3 (H3K9Ac), acetylation of lysine 8, 12 in H4 (H4K8Ac, H4K12Ac), and ubiquitylation of H2B (H2BK120u1) [26]. On the other hand, trimethylation of H3 at lysine 9 (H3K9me3), lysine 27 (H3K27me3) and ubiquitylation of histone H2A on lysine 119 (H2AK119u1) mark promoter regions of repressed genes [26].

Histone posttranslational modifications are regulated by the coordinated action of two groups of chromatin modifying enzymes, (i) “writers” including methyltransferases which add methyl marks and acetyl transferases that add acetyl marks as well as (ii) “erasers” including demethylases that remove methylation marks and deacetylases that remove acetylation marks. Histone acetylation is a highly dynamic modification regulated by the opposing action of two enzyme families, histone acetyltransferases (HATs) including MYST, GNAT and CBP/p300 families and histone deacetylases (HDACs) [27,28]. Acetyl marks neutralize positive charges on lysine amino acids that lead to the disruption of stabilizing electrostatic interactions within histone tails and aid in transcriptional activation, whereas removal of acetyl marks restore the positive charge on lysine residues, potentially stabilizing the local chromatin architecture [3]. Unlike acetylation, histone methylation does not alter the charge of a histone protein. Instead, histone methylation adds complexity to the transcriptional control of histones over gene expression as lysine residues may be mono-, di- or tri-methylated with increasing number of methyl groups generally correlating with higher levels of activation or repression depending on the lysine residue methylated [3,29,30]. Interestingly, histone lysine methyltransferase (HKMT) enzymes function in a relatively specific fashion by methylating the appropriate lysine to a specific degree. For example, the enzyme SET7 only mono-methylates H3K4 whereas MLL enzymes add additional methyl groups at lysine 4, resulting in di- (H3K4me2) and tri- (H3K4me3) methylation [26]. SUV39H1, the first HKMT to be identified, specifically targets H3K9 trimethylation [31], whereas Enhancer of zeste homolog 2 (EZH2), the enzymatic catalytic subunit part of the large Polycomb repressive complex 2 (PRC2) complex methylates K27 of the histone H3 (H3K27me3) [32].

H3K4me3 is considered a hallmark of active genes and is distributed along the promoter and TSS regions [33,34]. Providing a counterbalance, the histone modification marks H3K27me3 and H3K9me3 are hallmarks of repressive chromatin and are localized at silent gene loci, with H3K27me3 associated with facultative heterochromatin formation [35,36] and H3K9me3 involved in constitutive heterochromatin formation [37]. In addition to their association with distinct transcriptional states, posttranslational histone modifications exhibit “cross talk” either in *cis* positions on the same histone tail or in *trans* locations between adjacent histones, through which individual modifications reinforce or even reverse the effects of existing histone modifications. Moreover, other epigenetic regulators including those from the DNA methylation and RNAi machinery as well as non-coding RNAs also take part in this cross talk with histone modifications, leading to sequential recruitment of ATP-dependent chromatin remodeling complexes. For example, the stimulation of H3K4me3 and H3K79me3 marks by H2BK120u1 is a well-studied pathway for positive histone cross talk and is conserved in mammals [38]. Similarly, several studies have revealed that H3K4me3 promotes H3/H4 acetylation through recruitment of Histone Acetyl Transferases (HATs) [39], and both H3K4me3 and H3/H4 acetylation marks coexist at promoters and TSS of active genes [26].

## 5. Histone Modifications during Development

Transcriptional regulation of genes involved in mammalian development and organogenesis is intimately associated with histone posttranslational modifications. Promoter regions and enhancers of developmentally regulated genes in human and mouse embryonic stem cells (ESCs) exhibit a “bivalent” chromatin signature comprising both repressive H3K27me3 marks and active H3K4me3 marks [40,41]. Gene promoters marked as “bivalent” are believed to be repressed in ESCs but poised for activation in response to environmental and signaling cues allowing for cell differentiation to alternative fates during subsequent development and differentiation. As an example, for the repression of significant gene clusters in ES cells, the developmentally regulated *HOX* gene clusters and the inactive X chromosome are marked exclusively with H3K27me3 marks in ES cells [42,43]. More recently, a bipartite signature comprising active H3K27ac marks on promoters but repressive EZH2-dependent H3K27me3 marks on gene bodies has been revealed in several developing cell types including ES cells. This bipartite signature provides for rapid stimulus-induced transcriptional responses during development [44].

Dynamic changes in histone modifications also accompany the waves of DNA methylation and demethylation during PGC reprogramming and embryonic development [40,45,46,47]. Epigenetic reprogramming in nascent PGCs observed at E8.5 is characterized by a substantial loss of H3K9me2 and DNA methylation marks with a concomitant increase in several other histone marks including H3K27me3, H3K4me2 and H3K4me3 among others [48]. At E11.5, the repressive marks H3K9me3, H3K27me3 and the active H3K9Ac mark are lost, setting the stage for complete epigenetic reprogramming of the PGC genome [14].

In early mouse embryos, the active H3K4me3 mark is lost in the paternal genome after fertilization but is re-established during zygotic gene activation [49,50]. The paternal genome in zygotes also lack H3K9me3 and H4K20me3 marks [51,52]. Interestingly, during early embryogenesis, the repressive H3K27me3 marks are intergenerationally inherited from the maternal genome since the transition from morula to inner cell mass (ICM) and trophectoderm (TE) stages is accompanied by global erasure of H3K27me3 marks in the paternal genome and a selective depletion of H3K27me3 in the maternal genome [53].

Several studies have implied a functional relationship and mutual interdependence between the DNA methylation machinery and histone modifications in the regulation of gene transcription, especially in cancers. There have been several experimental studies demonstrating the effects of DNA methylation on histone modification changes and vice versa. Studies in mice have demonstrated that CpG methylation triggers a cascade of events beginning with the recruitment of MeCP1/MeCP2 proteins, which through their association with histone deacetylase complexes (Sin3, HDAC1 and HDAC2) lead to histone deacetylation or alternatively lead to H3K9 methylation via the SUV39H1 histone methyltransferase [54,55,56]. Similarly, the use of DNA demethylating agents like 5-aza-2′-deoxy-cytidine (5-aza-dC) activates the *RASSF1A* gene by inducing promoter histone acetylation [57]. While these studies point to DNA methylation as an initial event directing histone modifications, other reports have conversely demonstrated a dependence of the DNA methylation machinery on histone modifications. For example, the DNA methyltransferases DNMT3A/DNMT3B directly interact with the histone methyltransferases SUV39H1 and SETDB1 in mammals, suggesting that the de novo DNA methylation machinery may at least in part rely on pre-existing histone lysine methylation marks for recognizing chromatin substrates [58,59,60]. Another example for histone modification as the primary epigenetic event influencing the status of DNA methylation has been provided by the HDAC inhibitors (HDACi) valproate and Trichostatin A (TSA), suggesting that histone acetylation functions to protect against DNA methylation [61,62,63]. The interplay between these two epigenetic modifications is also well documented in *Neurospora*, where mutations in *dim-5*, the gene encoding histone H3K9 methyltransferase, abolished all CpG methylation, resulting in a reactivation of DNA methylation silenced genes [64].

## 6. Chromatin Remodeling Complexes as Regulators of Gene Expression and Development

Another group of epigenetic modifiers that affect DNA accessibility during development and differentiation are the ATPase subunit containing chromatin remodeling complexes, such as SWI/SNF (switching defective/sucrose nonfermenting), CHD (chromodomain helicase DNA-binding), ISWI (imitation switch), and INO80 (inositol requiring 80) complexes that rely on ATP catalytic energy to exchange histones and to slide or evict nucleosomes. These multi-component ATP dependent remodelers therefore directly affect gene expression through their impact on the assembly and dissolution of chromatin [65,66]. The ATPase subunits of these complexes are encoded by approximately 30 genes in mammals [4]. Some of these chromatin remodeling complexes are cell-type specific and developmental-stage specific. For example, the Brahma-associated factor (BAF) complex, a member of the SWI/SNF family, has been reported to form tissue-specific BAF complexes through interactions with a variety of transcription factors, resulting in several context-dependent functions [67,68]. Apart from their chromatin remodeling activities, these complexes have been attributed several additional functions beyond transcription, including a role in telomere regulation and chromosome segregation for the INO80 complex [69], and a function in the maintenance of higher order structure of the *D. melanogaster* X chromosome for the ISWI complex [70].

The ISWI family of ATP-dependent chromatin remodeling complexes were first identified in Drosophila where they play an important role in regulating higher-order chromatin structure with *Iswi* loss-of-function mutations resulting in global decondensation of mitotic chromosomes [69,71]. In mammals, the core ISWI complexes are composed of functionally distinct ATPases, SNF2H or SNF2L, which are components of different remodeling complexes facilitating a wide range of cellular functions ranging from transcription control, regulation of chromatin structure, DNA replication through heterochromatin and chromosome segregation [72].

The CHD family of chromatin-remodeling complexes comprise nine chromodomain-containing members (CHD1-9) and are broadly classified into three subfamilies based on their constituent domains (subfamily I to III) [73]. The subfamily I member, CHD1 is equipped to specifically recognize and bind the active chromatin associated H3K4me3 histone modification resulting in the recruitment of post-transcriptional initiation factors and transcriptional activation [74,75]. In mammals, the subfamily II members and core ATPases, CHD3 and CHD4 are subunits of NURD (nucleosome-remodeling and histone deacetylase) complexes, which contain HDACs and function as transcriptional repressors [76].

The INO80 family of chromatin remodelers includes INO80 and SWR1 ATPase containing large multi-subunit complexes which demonstrate in vitro nucleosome-remodeling activity and play important in vivo roles in transcriptional regulation [77]. The Swr1 complex in yeast, and the SWR1 homologues, p400 and SRCAP in mammals are required for deposition of the histone variant H2A.Z (Htz1 in yeast) into chromatin by replacing H2A [78,79]. H2A.Z is conserved from yeast to mammals and is essential for D. melanogaster and mouse development and is responsible for proper lineage commitment during mouse ESC differentiation [80].

## 7. Chromatin Remodelers during Development

Mutations of genes encoding the ATPase subunit of various chromatin remodeling complexes often lead to severe developmental defects in the early embryo and are haplo-insufficient (both copies of gene are required for proper development). The SWI/SNF remodelers have widespread developmental functions and are crucial for proper embryonic development in all organisms [4]. For example, mice deficient in BRG1, an ATPase subunit of the SWI/SNF complex in mammals, exhibit lethality at the peri-implantation stage due to its essential role in the regulation of self-renewal and pluripotency of mouse embryonic stem cells [81]. In contrast, mice deficient in SNF2H, one of the two ISWI complex ATPases, die after embryonic implantation, suggesting a crucial role of higher-order chromatin structure regulation in development [82]. A SWI/SNF complex member in mouse ES cells called esBAF is characterized by the presence of the BRG1 ATPase instead of BRM and plays a crucial role in the maintenance of the core pluripotency transcriptional network [83].

CHD1 is a CHD subfamily I ATPase initially thought to be integral to transcriptional activity because of its ability to recognize and bind H3K4me3 histone marks [74,75]. However, CHD mediated transcriptional activation is not conserved across species and while *D. melanogaster* CHD1 is involved in gametogenesis [84] CHD1 depletion in mouse ES cells results in a loss of pluripotency whereas CHD1 mediated maintenance of the euchromatic state preserves lineage plasticity [85]. Another developmental defect attributed to the CHD family of chromatin-remodeling complexes is the human CHARGE syndrome, a developmental malformation characterized by a severe retardation of growth and development, with *Chd7* heterozygous mice recapitulating several aspects of the human disease [86,87]. The *Drosophila* counterpart of *Chd7*, *Kismet* plays an important role in counteracting Polycomb repressive complex mediated transcriptional repression by recruiting the histone methyltransferases ASH1 and TRX to chromatin during development [88].

Several genome-wide studies conducted in ESCs have highlighted the essential role of chromatin remodeling complexes in ESC self-renewal, pluripotency and morphology, including the NURD complexes, TIP60-p400 complex, CHD1 and BAF complexes [89,90]. ChIP-seq studies have identified that the targets of the esBAF complex (SWI/SNF-like complexes in ESCs) overlap extensively with targets of the key pluripotency related transcription factors, OCT4, SOX2, NANOG, STAT3 and SMAD1, directly linking chromatin remodeling with maintenance of ‘stemness’ in ESCs [91,92].

## 8. Conclusions

In conclusion, this review has highlighted some of the powerful effects of key epigenetic factors, including DNA methylation, histone modifications, and chromatin remodeling complexes in the control of transcription and morphogenesis during mouse and human development.

## Data Availability

Not applicable.
